# Analysis of ASAS health index and its influencing factors in ankylosing spondylitis: a prospective study based on the population of Chaoshan region

**DOI:** 10.3389/fmed.2024.1499798

**Published:** 2025-01-08

**Authors:** Aikang Li, Rongji Liang, Liangbin Wu, Minghua Cai, Jiayou Chen, Yao Gong, Shaoyin Zeng

**Affiliations:** ^1^Shantou University Medical College, Shantou, China; ^2^Shenzhen University Medical College, Shenzhen University, Shenzhen, China; ^3^First Affiliated Hospital of Shantou University Medical College, Shantou, China

**Keywords:** ankylosing spondylitis, assessment of spondyloarthritis international society health index, quality of life, influencing factors, Chaoshan

## Abstract

**Objective:**

This study aimed to evaluate the health-related quality of life (HRQoL) in ankylosing spondylitis (AS) patients in the Chaoshan region and identify factors influencing the ASAS Health Index (ASAS-HI) to enhance comprehensive AS treatment strategies.

**Methods:**

A survey of ASAS-HI was conducted on 82 AS patients from the rheumatology outpatient department of the First Affiliated Hospital of Shantou University Medical College. The Bath Ankylosing Spondylitis Global Score (BAS-G) assessed overall health status, the Ankylosing Spondylitis Quality of Life Questionnaire (AS-QOL) evaluated quality of life, the Bath Ankylosing Spondylitis Disease Activity Index (BASDAI) measured disease activity, and the Bath Ankylosing Spondylitis Functional Index (BASFI) assessed functional difficulties. Inflammatory markers and patient data were collected, and univariate/multivariate logistic regression analyses were used to explore influencing factors of ASAS-HI.

**Results:**

The mean ASAS-HI score was 3.52 ± 3.12. ASAS-HI was positively correlated with BASDAI (*r* = 0.478, *p* < 0.001), ASDAS-CRP (*r* = 0.406, *p* < 0.001), BASFI (*r* = 0.338, *p* < 0.002), and BAS-G (*r* = 0.335, *p* < 0.002). Patients with ASDAS-ES*R* ≥ 2.1, ASDAS-CRP ≥ 2.1, and spinal tenderness had significantly higher ASAS-HI scores than others (*p* < 0.001). Spinal tenderness and radiographic grading were identified as key influencing factors.

**Conclusion:**

ASAS-HI is significantly impacted by disease activity and functional limitations. Early assessment of ASAS-HI is crucial for optimizing disease management in AS patients.

## 1 Introduction

Ankylosing spondylitis (AS) is a chronic progressive inflammatory autoimmune disease that affects the sacroiliac joints and the axial skeleton, and sometimes peripheral joints, periarticular tissues, and internal organs. As the disease progresses, deformities and stiffness of the spine can occur (1). Young men are the most common population affected by AS. In the Shantou area of Chaoshan, the prevalence of AS is 0.3% (2). Symptoms such as low back pain, morning stiffness, and fatigue may appear at the onset of the disease. Additionally, manifestations of enthesitis, such as heel pain, can also occur (3). Most patients are at the beginning or peak of their careers, and these symptoms can significantly impact their daily work efficiency and quality of life, leading to loss of social and familial productivity (4–6).

Currently, tools used to assess spondyloarthritis, such as the Bath Ankylosing Spondylitis Disease Activity Index (BASDAI), Ankylosing Spondylitis Disease Activity Score (ASDAS), and other commonly employed scales, primarily focus on specific aspects of health, such as pain, disease activity, and physical function, and are often utilized to evaluate physical performance and health-related quality of life (HR-QoL) (7). These tools, which were originally developed based on clinicians’ experiences with minimal input from patients, primarily measure the direct effects of the disease but fail to comprehensively address broader aspects of patient well-being, including overall quality of life, economic burden, and social relationships. Consequently, the limitations faced by patients with AS in daily activities or fulfilling social role obligations remain inadequately assessed. To overcome these shortcomings, the Assessment of Spondyloarthritis International Society Health Index (ASAS HI) was developed as a more holistic health assessment tool. The ASAS HI is a health index developed for AS patients based on the core set of the International Classification of Functioning, Disability and Health (ICF), aiming to evaluate the actual life status of ankylosing spondylitis patients (8, 9). ASAS HI includes 17 items covering aspects such as pain, emotional function, sleep, sexual function, activity, self-care, community life, and employment. Its reliability and validity have been confirmed (10). Therefore, applying ASAS HI for patient assessment can better assess the real-life quality of life of AS patients.

Previous research has demonstrated the strong convergent and discriminative validity of the Assessment of Spondyloarthritis International Society Health Index (ASAS-HI) with established measures such as the Bath Ankylosing Spondylitis Disease Activity Index (BASDAI) and the Ankylosing Spondylitis Disease Activity Score-C-Reactive Protein (ASDAS-CRP), highlighting its potential for routine clinical application (11). However, few studies have comprehensively evaluated its correlation with patient-reported outcome indicators such as mood disturbances, fatigue, and disease activity in clinical settings. Additionally, the relative importance of certain items in the ASAS-HI, such as using the toilet, driving, and hair washing, varies significantly between geographical regions due to cultural differences. Therefore, this study aims to explore the quality of life and influencing factors of AS patients in the Chaoshan region. By conducting multiple regression analysis on the correlations between various assessment tools, patient examinations, laboratory tests, and the ASAS-HI, the study seeks to provide guidance for developing personalized comprehensive treatment plans.

## 2 Methods

### 2.1 Subjects and inclusion criteria

This cross-sectional study included 82 AS patients recruited from the rheumatology outpatient department of the First Affiliated Hospital of Shantou University Medical College from July 2019 to January 2020 as observation and follow-up subjects. Inclusion criteria: (1) Patients who meet the revised New York criteria for AS diagnosis in 1984 (12), and all patients were diagnosed with AS by medical history and imaging examination; (2) Patients who are informed about the content and purpose of this study and sign an informed consent form. Exclusion criteria: (1) Patients with concomitant severe cardiac, hepatic, renal, or other important organ and blood, endocrine system lesions or history; (2) Patients who cannot cooperate to complete the self-assessment questionnaire and relevant examinations due to mental abnormalities or any other reasons.

### 2.2 Study design

#### 2.2.1 Socio-demographic characteristics

Record general patient information, including name, gender, age, marital status, occupation, education level, dietary habits, alcohol and tobacco use, and religious beliefs.

#### 2.2.2 ASAS hi

It includes 17 domains covering pain, maintaining a body position, moving around, toileting, energy and drive, motivation, sexual functions, driving, community life, handing stress, recreation and leisure, emotional functions, washing oneself, economic self-sufficiency, sleep, and handling stress. Patients self-assess each domain by selecting “agree,” “disagree,” or “not applicable.” A score of 1 is assigned for each domain marked as “agree,” with a total score calculated from the 17 domains. A score of 0 represents the best outcome, while 17 represents the worst (13).

#### 2.2.3 Disease assessment

1. Bath Ankylosing Spondylitis Global Score (BAS-G) (14): This assesses the overall condition of AS through a self-administered questionnaire, consisting of two questions. Patients are asked to mark their overall estimation of their condition over the past week and the past 6 months on a 10 cm visual analogue scale (VAS).
2. Bath Ankylosing Spondylitis Disease Activity Index (BASDAI) (15): Comprising six questions related to symptoms of fatigue, spinal pain, joint tenderness, local tenderness, and morning stiffness, BASDAI assesses patients’ scores for each question using a VAS. The average score is calculated, with a total range of 0–10 points, where 0 indicates the best and 10 indicates the worst.
3. Bath Ankylosing Spondylitis Functional Index (BASFI) (15): Based on prompts for the following 10 activities, patients are asked to mark the difficulty of completing these activities on a scale. The activities include putting on socks or close-fitting clothes, bending to pick up objects, reaching for items from a higher place, standing up from a chair without armrests, getting up from lying on the floor, standing for 10 min without discomfort, climbing 10–15 steps, looking backward, engaging in physical activities, and completing a day of household and work tasks. The average score is calculated, with 0 indicating the best and 10 indicating the worst.
4. C-reactive protein (CRP) levels were measured by fully automated dry chemistry method: normal range 0–10 mg/L; Erythrocyte sedimentation rate (ESR) was measured by fully automated erythrocyte sedimentation rate analyzer: normal range 0–15 mm/h; Ankylosing Spondylitis Disease Activity Score (ASDAS)-CRP (16): ASDAS-CRP = 0.121 × back pain score + 0.058 × morning stiffness duration score + 0.11 × patient global score + 0.073 × peripheral joint pain ÷ swelling 1score + 0.579 × ln(CRP + 1); Ankylosing Spondylitis Disease Activity Score (ASDAS)-ESR (17): ASDAS-ES*R* = 0.079 × back pain score + 0.069 × morning stiffness duration score + 0.113 × patient global score + 0.086 × peripheral joint pain ÷ swelling score + 0.293 × √ESR.

#### 2.2.4 Skeletal and muscular system activity assessment

1. Chest expansion (cm) (18): Standing upright, measure the horizontal distance between the fourth ribs using a scaled soft ruler, with the difference between maximum inspiration and maximum expiration. A difference of <2.5 cm is considered abnormal.
2. Schober’s test (cm) (19): Mark a point 10 cm above the midpoint of the line connecting the posterior superior iliac spines, then ask the patient to bend forward (while keeping both knees straight) to measure the maximum flexion of the spine. Normal mobility increases the distance by more than 5 cm, while those with spinal involvement increase the distance by less than 4 cm.
3. Finger-to-ground distance (cm) (20): Standing upright with legs and knees extended, bend forward, and move the index finger downwards along a vertical ruler placed in front of the foot. The distance from the ground is measured as the finger-to-ground distance.
4. Spinal mobility (degrees) (21): Includes lumbar spine flexion, extension, left lateral flexion, and right lateral flexion. Normal flexion is 45 degrees, extension is 35 degrees, and lateral flexion is 30 degrees on each side.
5. Spinal tenderness (22): The examinee adopts a seated position with slight forward bending. The examiner uses the thumb or index finger pad to sequentially press down from top to bottom on the spinous and transverse processes of the spine, as well as the paravertebral muscles. Normal findings should not produce tenderness.
6. Patrick’s test (Lower limb “4-letter test”) (23): The patient lies supine, with one knee flexed and the heel placed on the opposite extended knee. The examiner presses down on the flexed knee with one hand (while the hip joint is flexed, abducted, and externally rotated), while applying pressure to the opposite hip with the other hand. Pain in the contralateral sacroiliac joint is considered positive.

#### 2.2.5 Imaging index detection

**Figure 1:**
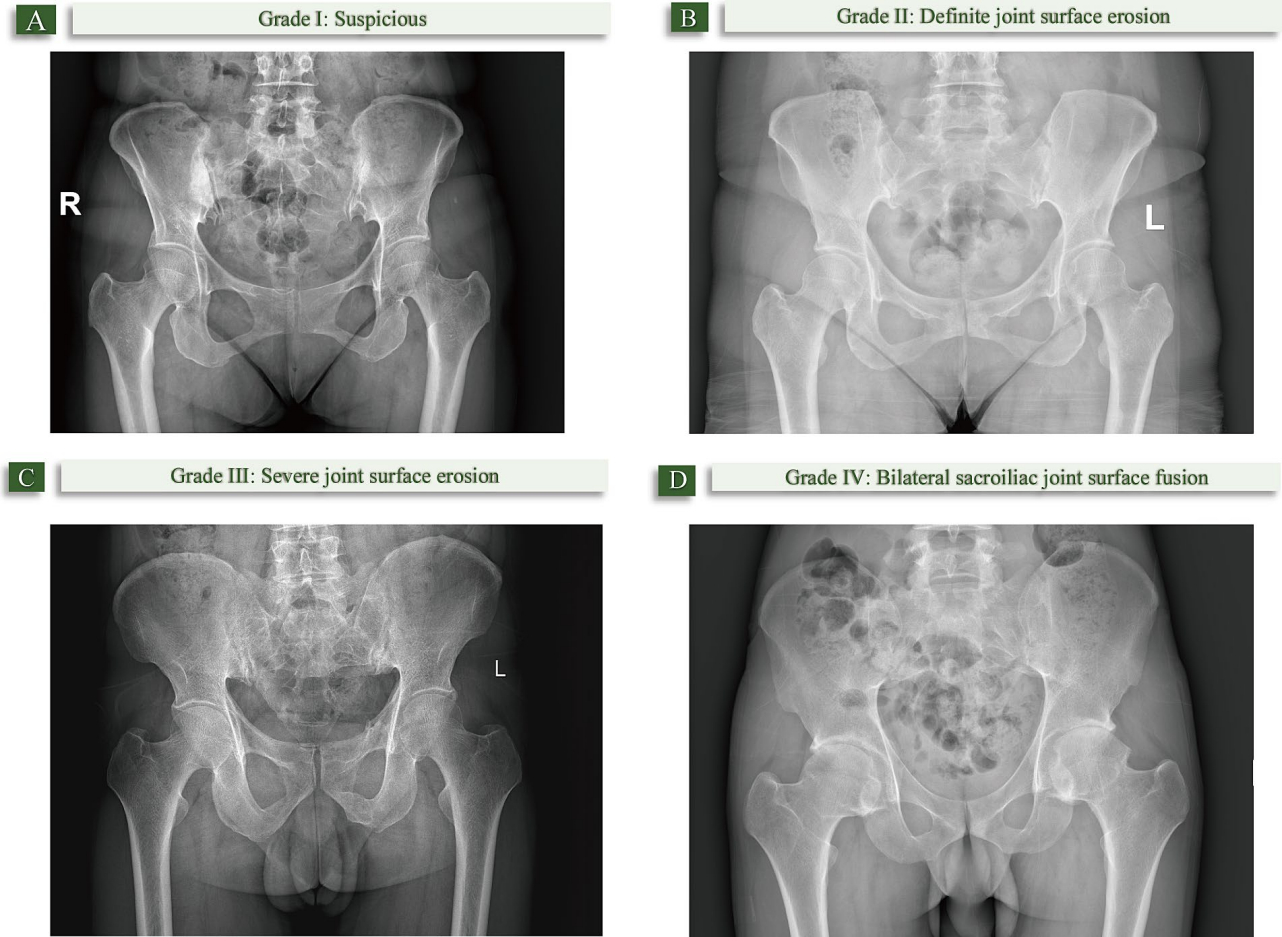
The degree of sacroiliitis on X-ray. **(A)** Grade I: Suspicious (joint surface roughness, small cystic changes, slight sclerosis); **(B)** Grade II: Definite joint surface erosion without significant joint space changes; **(C)** Grade III: Severe joint surface erosion, pseudo-widening/narrowing of joint space, or partial fusion; **(D)** Grade IV: Bilateral sacroiliac joint fusion.

The degree of sacroiliitis on X-ray is classified into 5 levels: Grade 0: Normal; Grade I: Suspicious (roughness of joint surface, small cystic changes, slight sclerosis); Grade II: Definite joint surface erosion, no significant change in joint space; Grade III: Severe joint surface erosion or secondary repair leading to pseudo-widening or narrowing of joint space, local fusion; Grade IV: Bilateral sacroiliac joint surface fusion (24). Diagnostic criteria for ankylosing spondylitis on imaging (25): Bilateral sacroiliitis Grade ≥ II, or unilateral sacroiliitis Grade III-IV (Figure 1).

### 2.3 Statistical analysis

Statistical analysis was performed using SPSS 20.0 software. Descriptive analysis was used for general data. Independent sample *t*-test was conducted to analyze whether there were differences in means between two groups, with results presented as mean ± standard deviation. Spearman correlation coefficient was calculated to assess the correlation between ASAS HI and other relevant disease indicators. Linear regression analysis was employed to identify predictors of ASAS HI. A *p*-value <0.05 was considered statistically significant.

## 3 Results

### 3.1 Baseline characteristics

This study included 82 AS patients who met the inclusion criteria. Among these patients, 64 were male (78.0%) and 18 were female (22.0%), with a mean age of 32.0 ± 11.3 years (range: 16–65 years). Educational attainment varied: 47 patients (57.3%) had a junior high school education or below, 8 patients (9.8%) had a high school education, and 27 patients (32.9%) had a college education or higher. Employment status revealed that 75 patients (91.5%) were employed, whereas 7 (8.5%) were unemployed. Marital status was nearly evenly distributed, with 40 patients (48.8%) being married and 42 (51.2%) unmarried. The average disease duration was 10.3 ± 7.1 years. Additionally, 70 cases (85.4%) tested positive for the HLA-B27 gene, while 12 cases (14.6%) tested negative (Table 1).

**Table 1:** General characteristics of ankylosing spondylitis patients.

Item		Data (*N* = 82)	
Age, years [Mean (SD)]		32.0 ± 11.3	
Disease duration, years [Mean (SD)]		10.3 ± 7.1	
Sex (*n*, %)	Male	64	78.0%
	Female	18	22.0%
Education (*n*, %)	Middle school or less	47	57.3%
	High school	8	9.6%
	College or more	27	32.9%
Marital status (*n*, %)	Single	42	51.2%
	Married	40	48.8%
Employment status (*n*, %)	Employment	75	91.5%
	Unemployed	7	8.5%
HLA-B27 (*n*, %)	Positive	70	85.4%
	Negative	12	14.6%

SD, standard deviation; HLA-B27, human leukocyte antigen-B27.

### 3.2 Comparison of ASAS HI scores according to disease activity status

ASAS HI score was 3.52 ± 3.12 (0–17). Scores of 0–5 were considered good in 60 cases (73.2%); 6–12 were considered moderate in 21 cases (25.6%); and 13–17 were considered poor in 1 case (1.2%). ASDAS has well-validated cutoff values, with ASDAS <2.1 indicating low disease activity and ASDAS ≥2.1 indicating high disease activity. ASAS HI scores were significantly higher in patients with ASDAS-CRP ≥2.1 (5.21 ± 3.52) compared to ASDAS-CRP <2.1 (2.65 ± 2.45) (*p* < 0.001); ASAS HI scores were also significantly higher in patients with ASDAS-ESR ≥2.1 (5.32 ± 3.80) compared to ASDAS-CRP <2.1 (2.59 ± 2.21) (*p* < 0.001); ASAS HI scores in patients with spinal tenderness (5.58 ± 3.52) were significantly higher than those without tenderness (2.90 ± 2.72) (Table 2).

**Table 2:** Comparing ASAS HI scores among different disease activity States (*n* = 82).

	ASDAS-CRP		ASDAS-ESR		BASDAI		Spinal tenderness	
Grade	<2.1	≥2.1	<2.1	≥2.1	<4	≥4	Negative	Positive
HI (X ± S)	2.65 ± 2.45	5.21 ± 3.52	2.59 ± 2.21	5.32 ± 3.80	3.22 ± 2.87	4.87 ± 3.89	2.90 ± 2.72	5.58 ± 3.52
*n*	54 (65.9%)	28 (34.1%)	54 (65.9%)	28 (34.1%)	67 (81.7%)	15 (18.3%)	63 (76.8%)	19 (23.2%)
*P*	<0.001		<0.001		>0.05		<0.001	

ASDAS-CRP, ankylosing spondylitis disease activity score - C-reactive protein; ASDAS-ESR, Ankylosing spondylitis disease activity score - erythrocyte sedimentation rate; BASDAI, bath ankylosing spondylitis disease activity index.

### 3.3 Correlation of ASAS HI with disease activity and musculoskeletal function

ASAS HI showed a strong correlation with BASDAI (*r* = 0.478, *p* < 0.001). Additionally, ASAS HI was positively correlated with ASDAS-CRP (*r* = 0.406, *p* < 0.001), pain (*r* = 0.369, *p* < 0.001), BASFI (*r* = 0.338, *p* < 0.002), BAS-G (*r* = 0.335, *p* < 0.002), ASDAS-ESR (*r* = 0.331, *p* < 0.002), and fingertip-to-floor distance (*r* = 0.273, *p* < 0.013). No correlation was found between ASAS HI and chest expansion or Schober test (*p* > 0.05) (Table 3).

**Table 3:** Correlation between ASAS HI and disease activity and musculoskeletal activity.

	ASDAS-CRP	ASDAS-ESR	BAS-G	BASDAI	BASFI	Pain	Finger-to-floor distance	Chest expansion	Schober’s test
*r*	0.406	0.331	0.335	0.478	0.338	0.369	0.273	0.001	0.052
*P*	<0.001	<0.002	<0.002	<0.001	<0.002	<0.001	<0.013	>0.05	>0.05

ASDAS-CRP, ankylosing spondylitis disease activity score based on C-reactive protein; ASDAS-ESR, ankylosing spondylitis disease activity score based on erythrocyte sedimentation rate; BAS-G, bath ankylosing spondylitis global score; BASDAI, bath ankylosing spondylitis disease activity index; BASFI, bath ankylosing spondylitis functional index.

### 3.4 Correlation between ASAS HI and radiographic grading

Bilateral sacroiliitis grade II was observed in 38 cases (46.3%), grade III in 29 cases (35.4%), and grade IV in 15 cases (18.3%). The ASAS HI for grade II bilateral sacroiliitis was 2.39 ± 2.62; for grade III, it was 4.48 ± 3.26 on average; and for grade IV, it was 4.53 ± 3.23. There was a weak correlation between ASAS HI and radiographic grading (*r* = 0.281, *p* < 0.002) (Table 4).

**Table 4:** The correlation between ASAS HI and radiographic grade.

	Radiographic grade		
	II	III	IV
*n*	38	29	15
HI (^−^X ± S)	2.39 ± 2.62	4.48 ± 3.256	4.53 ± 3.23
*r*	0.281		
*P*	<0.002		

### 3.5 Multiple linear regression analysis

Multiple linear regression was used to predict ASAS HI based on BAS-G, BASDAI, BASFI, ASDAS-CRP, ASDAS-ESR, and radiographic grading. The results showed that spinal tenderness and radiographic grading of sacroiliitis were positively correlated with ASAS HI (*β* = 1.828, *p* = 0.027; *β* = 0.957, *p* = 0.029). The regression model was statistically significant, *F* = 5.238, *p* < 0.001, adjusted *R*^2^ = 0.239. Spinal tenderness and radiographic grading were the main influencing factors of ASAS HI (Table 5).

**Table 5:** Linear regression of factors influencing ASAS HI.

	*β*	SE	*p*
ASDAS-CRP	0.568	0.772	0.464
ASDAS-ESR	−0.799	0.764	0.299
BAS-G	0.033	0.214	0.877
BASDAI	0.474	0.447	0.293
BASFI	0.368	0.274	0.184
Pain	−0.021	0.227	0.927
Finger-to-floor distance	0.015	0.029	0.599
Spinal tenderness	1.828	0.812	0.027
Radiographic grade	0.957	0.429	0.029

ASDAS-CRP, ankylosing spondylitis disease activity score based on C-reactive protein; ASDAS-ESR, ankylosing spondylitis disease activity score based on erythrocyte sedimentation rate; BAS-G, bath ankylosing spondylitis global score; BASDAI, bath ankylosing spondylitis disease activity index; BASFI, bath ankylosing spondylitis functional index.

## 4 Discussion

Previous studies have shown that chronic diseases are one of the most important factors affecting the quality of life of Chinese residents (26). AS, as a major chronic inflammatory rheumatic disease affecting young adults, is characterized by recurrent pain, fatigue, and functional impairment (27); as the disease progresses, the entire spine can become rigid from bottom to top, with late-stage manifestations including hip stiffness, severe spinal deformity, and kyphosis, which can lead to disability and impose significant pressure on patients and their families, severely affecting patients’ quality of life (28). The selection of items for the ASAS Health Index (HI) was conducted collaboratively among rheumatologists, healthcare professionals, and patients with AS from a core set of ICF variables. This process underscores the ASAS HI serves as a practical assessment of patients’ actual discomfort in daily life. Assessing the quality of life and social functioning of patients with chronic diseases can provide a basis for improving treatment strategies and evaluating treatment outcomes, as well as facilitating health education for patients, improving prognosis, and reducing the degree of social function impairment in patients (29). However, the factors influencing ASAS HI have not been clearly demonstrated so far. In the present study, we determined ASAS HI prospectively in a single cohort and identified several factors including various assessment tools and data from physical examinations, laboratory tests and imaging that are associated with ASAS HI. BASFI, BAS-G, ASDAS-CRP, ASDAS-ESR, fingertip-to-floor distance, hip joint pain severity, spinal tenderness, and radiographic grading of sacroiliitis were found to be correlated with HI scores.

### 4.1 The impact of functionality and ability on the quality of life of AS patients

The BASFI scale, developed for defining and monitoring functional capabilities in individuals with AS (30), is commonly applied in clinical practice to predict patients’ work performance and everyday functioning (31). In a recent study, Imke Redeker et al. demonstrated that physical function significantly influences the ASAS Health Index (HI) through a Bayesian network analysis that integrates expert knowledge and observational data (32). The BAS-G is a patient-centered assessment tool that provides a comprehensive insight into the impact of ankylosing spondylitis (33). Our study revealed a positive correlation between patients’ quality-of-life HI scores and their BASFI scores for ability and function (*r* = 0.338), consistent with previous research (34), as well as with their overall condition as measured by the BAS-G scale (*r* = 0.335). This indicates that patients’ susceptibility to fatigue, ability to engage in physical activities, and performance in daily functions significantly influence their quality of life. Furthermore, the diminished quality of life exacerbates patients’ discomfort with their current physical condition. However, the relatively low correlation coefficient (35) may be attributed to the fact that both the BAS-G and BASFI rely predominantly on the subjective evaluations of patients,which potentially limits the precision and reproducibility of the results. Fatigue has been shown to be one of the most significant factors affecting patients’ well-being (14). Adding direct fatigue measurement tools, such as the Fatigue Severity Scale, may provide deeper insights into the potential fatigue factors reflected in BASFI and BAS-G scores. This approach could help align quality-of-life assessments with disease management strategies more effectively. As an important non-pharmacological treatment for AS, rehabilitation exercises can effectively improve the life and work ability of patients. A meta-analysis reveals that various forms of exercise, including home-based exercise programs, swimming, Pilates training, and supervised exercises, have a positive effect on the BASFI across all selected studies. Furthermore, no heterogeneity was observed in the reduction of BASFI [−0.72 (−1.03; −0.40)] and the reduction of trials involving anti-TNF therapy [−0.81 (−1.25; −0.38)], thereby reinforcing the potential of exercise programs to improve physical function in patients with AS (36). Patients need to consciously engage in muscle strength exercises, spinal flexibility exercises, chest expansion exercises, etc., in their daily lives, and avoid joint-impact, explosive, and high-load exercises. They can participate in swimming, yoga, tai chi, and other sports (37, 38). However, most patients lack sufficient understanding of AS-related knowledge, do not understand the importance of functional exercise in preventing spinal dysfunction, and often have poor compliance with rehabilitation treatment. How to promote the widespread adoption of rehabilitation exercises, which are effective and simple, is worth further research.

### 4.2 The impact of disease activity on the quality of life of AS patients

Among the proposed indices to evaluate disease activity of AS, BASDAI is currently the most widely used in clinical trials and in daily practice as yet (39, 40). However, the BASDAI scale can present ambiguities in assessing disease activity due to its dependence on patients’ subjective self-assessment, which introduces certain limitations in its clinical use (41). In order to more systematically and objectively evaluate the disease activity of patients, this study introduced the Ankylosing Spondylitis Disease Activity Score (ASDAS) that reflects several aspects of disease activity and shows a strong correlation with both physician and patient assessments of the condition, including both ASDAS-CRP and ASDAS-ESR indices. ASDAS is advocated as the primary tool for assessing disease activity in axSpA. The 2022 ASAS-EULAR management update for axSpA highlights that the main objective of treatment is to optimize health-related quality of life by managing symptoms and inflammation, preventing further structural damage, and maintaining or restoring function and social participation (42). The analysis revealed that there was a certain correlation between the Health Index (HI) and BASDAI (*r* = 0.478), ASDAS-ESR (*r* = 0.331), and ASDAS-CRP (*r* = 0.406). Groups with higher disease activity showed greater mean ASAS HI scores compared to those with lower activity, suggesting that ASAS-HI effectively differentiates disease activity levels, a key factor in evaluating its clinical relevance. Furthermore, HI was positively correlated with the degree of lumbar, back, and hip joint pain (*r* = 0.369), as well as spinal tenderness and other disease activity indicators. Pain is one of the most direct effects of AS, results from either inflammation or neuropathic mechanisms (43).It significantly affects patients’ daily activities and contributes to a decline in quality of life. The clinical characteristics of AS include a long course, prolonged medication duration, and recurrent disease episodes, necessitating correct and regular medication and rehabilitation training to reduce disease activity (44). However, due to the long duration of drug use, severe adverse reactions, and high treatment costs, patients often have poor compliance, resulting in frequent disease activity, worsening condition, and affecting their quality of life. Therefore, in order to improve patient compliance, medical staff need to understand the patients’ economic conditions and daily lives, and adopt the most effective, least adverse reactions, and patient-friendly treatment plans. On the other hand, patients also need to follow the doctor’s arrangements, and family members should provide sufficient care to ensure that patients receive comprehensive support and care, in order to improve treatment compliance and quality of life. Besides, the difference in BASDI scores was not statistically significant, which may be related to the smaller number of patients with disease activity (BASDI≥4), and further investigation is needed to expand the sample size.

### 4.3 The relationship between spinal mobility and HI scores

The characteristic feature of AS is the gradual onset of sacroiliitis and fibrosis as well as ossification of various joints of the spine (45), with sacroiliac joints often being the first affected (46). As the condition progresses, the lesions continue to accumulate upward involving the lumbar, thoracic, and cervical vertebrae, eventually leading to spinal stiffness in the late stage (47). Stiffness, included in the ASAS core domains for assessment, contributes to pain, functional impairment, and reduced quality of life (48). This study utilized the Schober test, finger-to-floor distance, and chest expansion to measure spinal mobility in AS patients. The results showed a weak correlation between HI score and finger-to-floor distance (*r* = 0.273), while there was no statistically significant association with Schober test or chest expansion. Furthermore, the radiographic grading of sacroiliitis can reflect the severity of sacroiliac joint involvement, with more severe involvement leading to poorer mobility of the sacroiliac joints. HI score exhibited a weak correlation with the radiographic grading of sacroiliitis (*r* = 0.281), indicating that higher grading was associated with higher HI scores. Some studies (49–51) have shown that spinal mobility can affect the quality of life of patients, and there may be discrepancies with the results of this experiment, possibly due to the focus of the HI scale on measuring the fatigue level and functional abilities of patients, which requires further investigation.

Our study has some limitations. Firstly, our study focused exclusively on AS patients who met the modified New York criteria, which may limit the applicability of the findings to non-radiographic axSpA cases. Secondly, as this is a cross-sectional study, any conclusions drawn regarding causality should be considered provisional. Additionally, MRI of the sacroiliac joints and spine may serve as a potential marker for disease activity (52). However, this study did not include MRI evaluation criteria to define disease activity, which may have resulted in the inability to perform a more comprehensive assessment of the patients’ disease activity.

In summary, the quality of life of AS patients is related to their daily functional abilities and activities; moreover, disease activity also affects patients’ quality of life. Our findings may drive attention to pharmacotherapy and exercise rehabilitation exercises to reduce disease activity and enhance mobility and functionality in patients with AS. Additionally, healthcare professionals should tailor treatment plans to the individual circumstances of patients to improve compliance, while family members should provide sufficient support and care to enhance the overall quality of life for patients.

## Data Availability

The original contributions presented in the study are included in the article/supplementary material, further inquiries can be directed to the corresponding authors.
